# Hemodynamic, Oxygenation and Lymphocyte Parameters Predict COVID-19 Mortality

**DOI:** 10.3390/pathophysiology30030025

**Published:** 2023-08-02

**Authors:** Choirina Windradi, Tri Pudy Asmarawati, Alfian Nur Rosyid, Erika Marfiani, Bagus Aulia Mahdi, Okla Sekar Martani, Giarena Giarena, Esthiningrum Dewi Agustin, Milanitalia Gadys Rosandy

**Affiliations:** 1 Department of Internal Medicine, Faculty of Medicine, Airlangga University, Surabaya 60286, East Java, Indonesia; chylonly@gmail.com (C.W.); alfian-n-r-10@fk.unair.ac.id (A.N.R.); erika.marfiani@fk.unair.ac.id (E.M.); okla.sekar.martani-2019@fk.unair.ac.id (O.S.M.);; 2 Universitas Airlangga Hospital, Airlangga University, Surabaya 60115, East Java, Indonesia; 3 Department of Pulmonary and Respiratory Medicine, Faculty of Medicine, Airlangga University, Surabaya 60286, East Java, Indonesia; 4 Department of Internal Medicine, Faculty of Medicine, Brawijaya University, Malang 65145, East Java, Indonesia; milanitalia@ub.ac.id

**Keywords:** COVID-19, infectious disease, scoring, prediction, mortality

## Abstract

The mortality of COVID-19 patients has left the world devastated. Many scoring systems have been developed to predict the mortality of COVID-19 patients, but several scoring components cannot be carried out in limited health facilities. Herein, the authors attempted to create a new and easy scoring system involving mean arterial pressure (MAP), PF Ratio, or SF ratio-respiration rate (SF Ratio-R), and lymphocyte absolute, which were abbreviated as MPL or MSLR functioning, as a predictive scoring system for mortality within 30 days for COVID-19 patients. Of 132 patients with COVID-19 hospitalized between March and November 2021, we followed up on 96 patients. We present bivariate and multivariate analyses as well as the area under the curve (AUC) and Kaplan–Meier charts. From 96 patients, we obtained an MPL score of 3 points: MAP < 75 mmHg, PF Ratio < 200, and lymphocyte absolute < 1500/µL, whereas the MSLR score was 6 points: MAP < 75 mmHg, SF Ratio < 200, lymphocyte absolute < 1500/µL, and respiration rate 24/min. The MPL cut-off point is 2, while the MSLR is 4. MPL and MSLR have the same sensitivity (79.1%) and specificity (75.5%). The AUC value of MPL vs. MSLR was 0.802 vs. 0.807. The MPL ≥ 2 and MSLR ≥ 4 revealed similar predictions for survival within 30 days (*p* < 0.05). Conclusion: MPL and MSLR scores are potential predictors of mortality in COVID-19 patients within 30 days in a resource-limited country.

## 1. Introduction

Still ongoing and expanding worldwide, up until 2023, the severe acute respiratory syndrome coronavirus 2 (SARS-CoV-2) pandemic continues in Indonesia. The Indonesian government has reported that, as of 2023, the number of confirmed COVID-19 patients had reached more than 6 million cases, with the number of mortalities reaching more than 160 thousand cases [1]. The clinical spectrum presented by patients infected with coronavirus 2 (COVID-19) varies from asymptomatic, mild upper respiratory tract infection to severe viral pneumonia with ARDS to multi-organ failure, the leading cause of mortality [2].

A large-scale observational study conducted by ISARIC (International Severe Acute Respiratory and Emerging Infection Consortium) estimates that the case fatality rate (CFR) of COVID-19 cases in hospitals has reached 33% [3]. Utilizing prognostic scores to determine patient-based therapy is essential to reducing COVID-19 mortality. Previously, there were several validated prognostic scores in the pre-COVID-19 period used in the acute setting, such as CURB-65 (Confusion, Urea, Respiratory Rate, Blood Pressure, and Age Above or Below 65 Years), NEWS2 (National Early Warning Score 2), and qSOFA (Quick Sequential Sepsis-Related Organ Failure Assessment). Since there is no specific prognostic score model for COVID-19, they have also often been used during this pandemic [4]. Nonetheless, the validity of the prognostic score for COVID-19 still needs to be improved. Also, the ability to predict the risk of mortality is still limited.

Numerous studies on specific prognostic models to predict deterioration and mortality in COVID-19 patients continue to progress, especially in developed countries [5,6,7,8,9,10,11,12]. Differences in the quality and quantity of health facilities, population characteristics, and comorbidities between developed countries and developing countries, such as Indonesia, may affect various predictive models of risk factors for death in hospitalized COVID-19 patients [12]. In Indonesia, particularly in limited healthcare facilities in remote areas, an easy and convenient scoring system to determine the severity of COVID-19 pneumonia is a crucial tool to avoid referral delays to more advanced healthcare facilities. Identifying patient-specific risk factors through easy, rapid, and accurate prognostic scores can assist clinicians in providing more aggressive therapy with limited facilities in Indonesia [13].

Parameters such as PaO_2_/FiO_2_ ratio, SaO_2_/FiO_2_ ratio, and respiratory rate have the potential to predict mortality in COVID-19. The clinical significance of the PaO_2_/FiO_2_ ratio and the SaO_2_/FiO_2_ ratio is that they represent the oxygen distribution to tissues, which, in COVID-19, is affected so much and thus leads to respiratory distress [7]. These parameters can also be used for other viral diseases, like dengue shock syndrome [8]. The validity study of the 4C mortality score by ISARIC also involved respiratory function parameters such as oxygen saturation and respiratory rate [14,15]. MAP (Mean Arterial Pressure) and heart rate represent cardiovascular functions that can be disrupted due to the systemic inflammatory process in COVID-19 and are significantly associated with increased mortality [16,17].

Several publications on SOFA, qSOFA, NEWS2, and APACHE discussing prognostic markers for COVID-19 disclosed various results in predicting mortality. Our previous study in Indonesia comparing SOFA, qSOFA, NEWS2, and APACHE showed that these scores precisely predict COVID-19 mortality after day 5 [18]. Unfortunately, as mentioned earlier, some components of the scoring system are not applicable in certain healthcare facilities in Indonesia, such as blood gas analysis.

The authors attempted to develop a simple prognostic score model, which we shorten to MPL (MAP, PaO_2_/FiO_2_ (PF) ratio, and lymphocyte absolute) or MSLR (MAP, SaO_2_/FiO_2_ (SF) ratio, lymphocyte absolute, respiration rate (RR)) to foretell the 30-day mortality of COVID-19 patients. These will help clinicians identify patients with poor prognoses at the beginning of diagnosis and regulate therapy management, especially in limited healthcare settings.

## 2. Materials and Methods

### 2.1. Study Design, Definition, and Sample Selection

We conducted a prospective cohort study, collecting data from 132 COVID-19 patients hospitalized in a high care unit (HCU) at a secondary referral hospital in Surabaya from March to November 2021. Inclusion criteria were patients with moderate to severe COVID-19 along with complete medical records consisting of the measurement of blood pressure, pulse, respiratory rate, peripheral O_2_ saturation, oxygen supplementation, complete blood count, and blood gas analysis on admission. Patients must then undergo complete monitoring for the next 30 days. Patients with mild symptoms, incomplete medical records, or discharge against medical advice (DAMA) within less than 30 days of hospitalization were excluded from the study.

Based on Indonesia’s COVID-19 management guidelines, patients with moderate disease have clinical symptoms of pneumonia (fever, cough, shortness of breath, rapid breathing) but do not exhibit severe pneumonia-related symptoms, such as oxygen saturation levels lower than 93% in room air. Patients with a severe illness have clinical indicators of pneumonia (fever, coughing, shortness of breath), a respiratory rate of more than 30 breaths per minute, significant respiratory distress, or a SpO_2_ of less than 93% on room air. Patients with critical illnesses experience acute respiratory distress syndrome (ARDS), sepsis, and septic shock [18].

**Figure 1:**
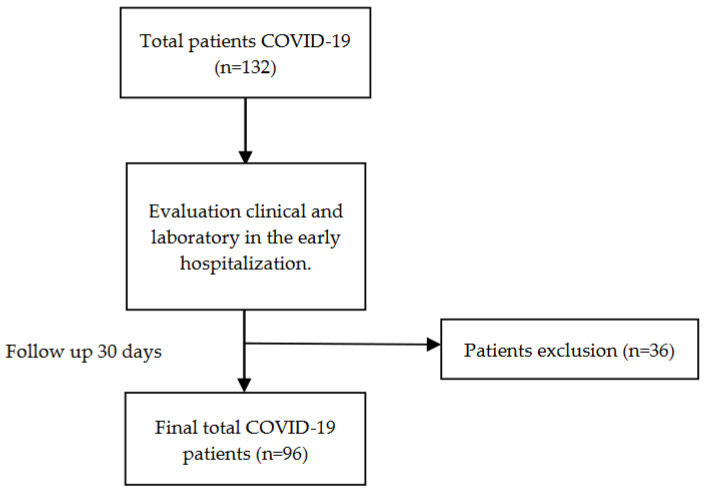
Selection of patients.

We had a follow-up for 30 days and evaluated clinical and laboratory outcomes. Of the 132 patients, 96 were successfully followed up for 30 days, while 36 had incomplete clinical and laboratory data (see Figure 1). By collecting 96 patients’ data in total, we were able to design a predictive score of 30 days’ mortality with 3–4 variables. Variables taken initially on the day of admission were: MAP, PF Ratio, SaO_2_/FiO_2_, SF Ratio-R, and absolute lymphocytes. MAP was measured on the first day (D-0), and we evaluated the preliminary data on the prognosis of death at 30 days. We categorized the critical value of each variable as follows: (i) MAP < 75 [19], (ii) PF Ratio < 200 or SF Ratio < 200 [20,21], (iii) absolute lymphocyte count (ALC) < 1500 [22], and (iv) respiration rate of 24 breaths/min [23]. Eventually, we named those predictive scores MPL (MAP, PF Ratio, Lymphocyte Absolute) and MSLR (MAP, SF Ratio, Lymphocyte Absolute, Respiration Rate).

The study was conducted in accordance with the Declaration of Helsinki and approved by the Institutional Review Board of Universitas Airlangga (172/KEP/2020).

We compared these two predictive scores in view of the role of respiratory function and oxygenation, which could be measured by blood gas analysis, in predicting mortality. Given that not all hospitals in Indonesia have complete laboratory facilities.

### 2.2. Statistical Analysis

We analyzed the data using SPSS version 24 (Chicago, IL, USA; RRID: SCR_002865), an open-access alternative. Baseline characteristics of subjects are described as the mean ± standard deviation, or median number. We scored MPL and MSLR based on bivariate analysis and multivariate logistic regression. Bivariate analysis was carried out by comparing two groups, critical and non-critical. The analysis then continued with multivariate logistic regression. Mortality thresholds from each score MPL and MSLR were performed; the receiver-operating curve (ROC) analysis associated with the area under the curve (AUC) was used to analyze the optimal parameter value of the laboratory to predict the progression of mortality in the study group. Excellent AUC lies between 0.9 and 1; good if 0.8 < AUC < 0.9; moderate if 0.7 < AUC < 0.8; poor if 0.6 < AUC < 0.7; and failed if 0.5 < AUC < 0.6. Our Kaplan–Meier graphic shows the survival of death in 30 days.

## 3. Results

The baseline demographics and clinical characteristics of the ninety-six included patients are summarized in Table 1. Overall, 76% of patients admitted experienced severe COVID-19, with males and females accounting for 51% and 49%, respectively. 44.8% of the patients died, with a mean treatment duration of around 14 days. Diabetes mellitus (40.6%) and hypertension (33.3%) were the most frequent comorbidities.

The effects of MAP, oxygen saturation, PF ratio, respiration rate, and absolute lymphocyte count as significant independent predictors of outcomes were analyzed to generate coefficients used in formulating the numerical scoring model.

Fifteen patients had MAP < 75 mmHg; 37 and 36 patients had PF ratios < 200 and SF ratios < 200, respectively; 34 patients had ALC < 1500 and 35 patients had RR 24x/min. Based on bivariate analysis, all variables differed significantly (*p* < 0.05). Furthermore, based on multivariate regression logistic analysis, all MPL model variables differed significantly (*p* < 0.05), while in MSLR, only absolute lymphocyte and respiration rate revealed significant differences (*p* = 0.009 and *p* = 0.03). The scores resulting from each MPL and MSLR variable are presented in Table 2. The point for each category will be either 0, 1, or 2, depending on the cut-off point (see Table 3).

**Figure 2:**
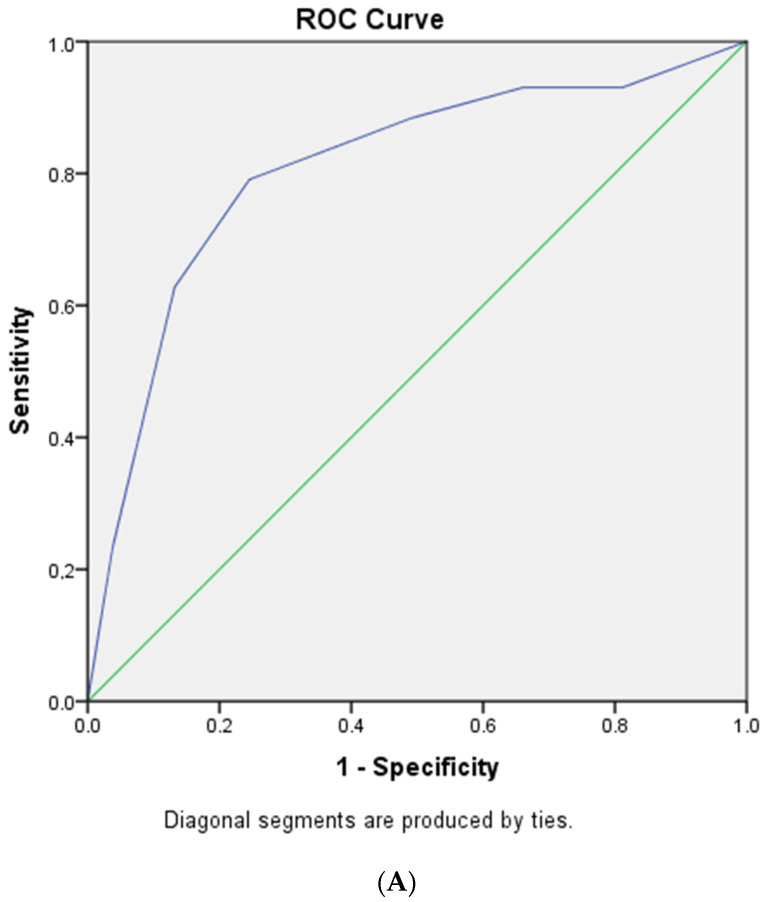

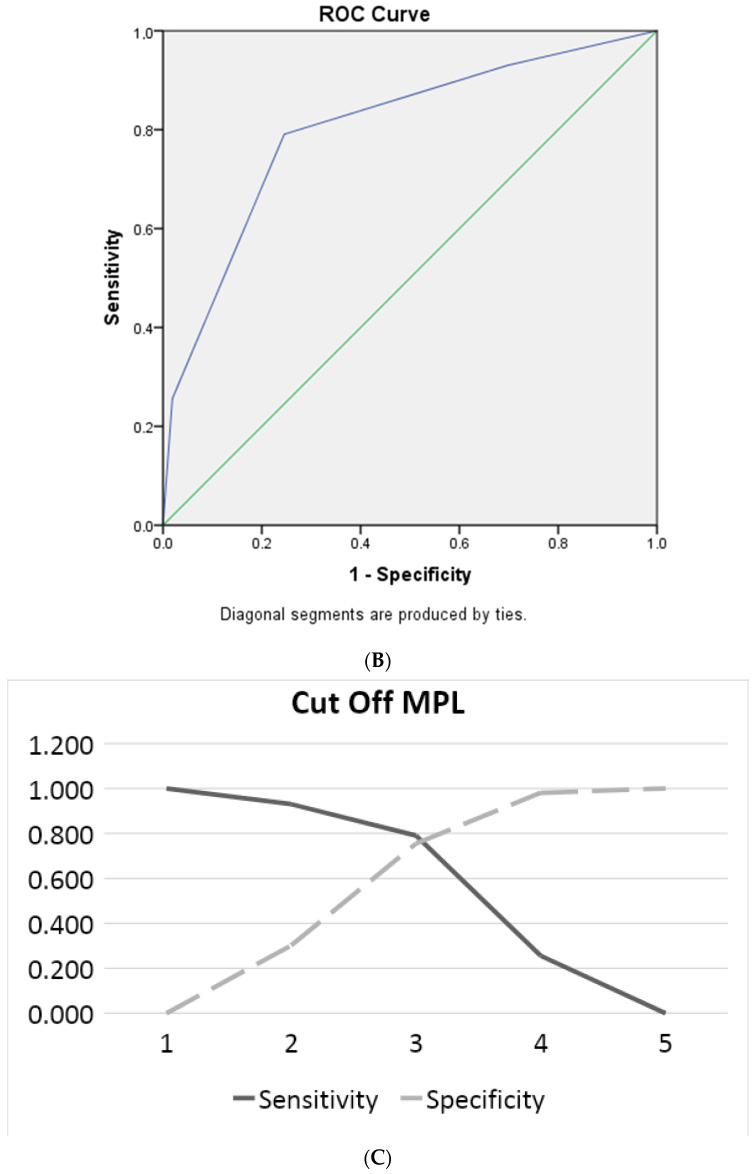

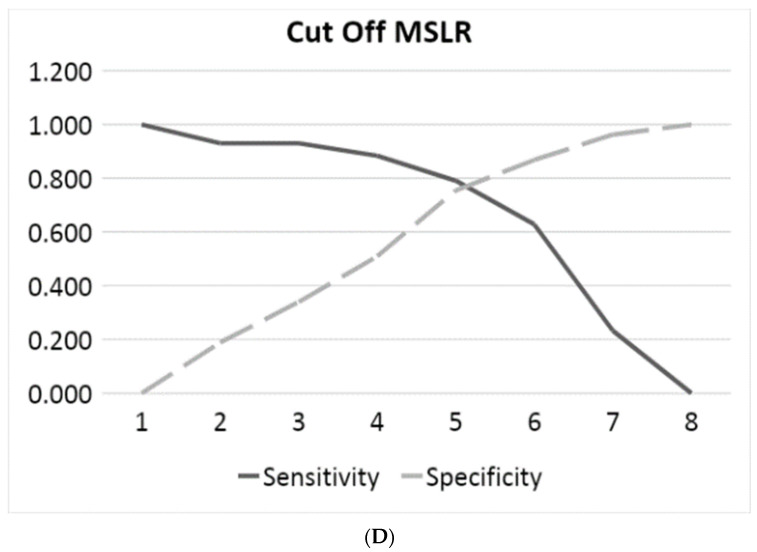
(**A**) AUC MPL score to predict mortality in COVID-19 patients; (**B**) AUC MSLR score to predict mortality in COVID-19 patients; (**C**) cutoff for MPL score; (**D**) cutoff for MSLR score. AUC, the area under the curve; MPL, MAP, PF Ratio, and Lymphocyte absolute; MSLR, MAP, SF Ratio, Lymphocyte absolute, and Respiration rate. The blue line refers to the sensitivity, while the green line refers to the area under the curve.

**Figure 3:**
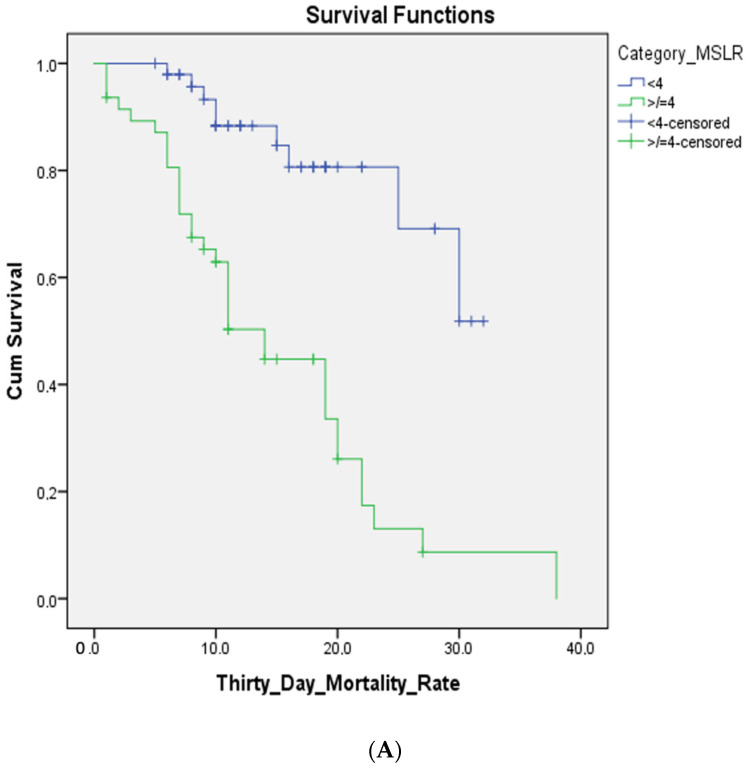

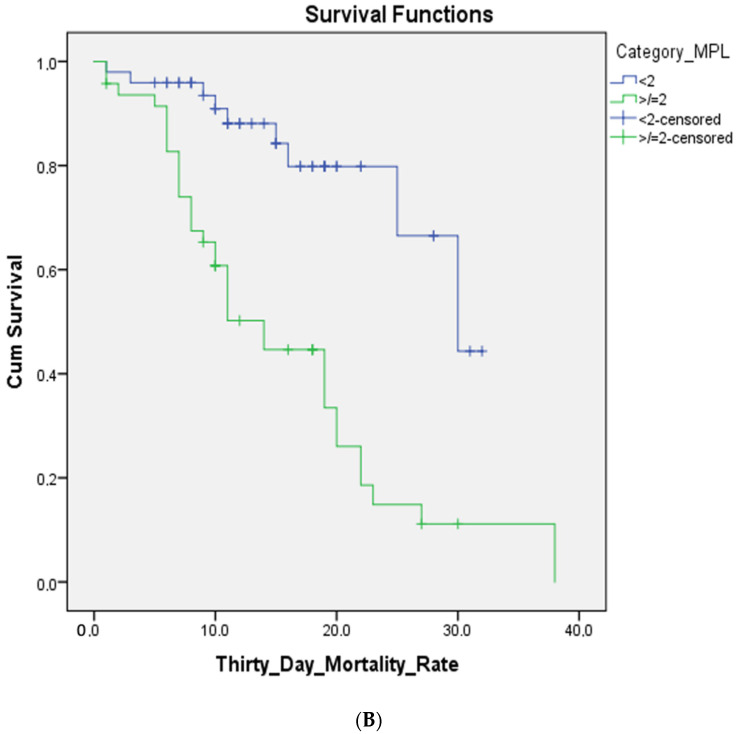
(**A**) Survival 30-day mortality rate from MPL score; (**B**) survival 30-day mortality rate from MSLR score.

Based on multivariate analysis, the cut-off points for each variable were obtained. Each of the examination parameters above produces a score that can be calculated as a predictor. MPL is an acronym for MAP (<75 mmHg), PF ratio (<200), ALC (<1500/μL), and MSLR is an acronym for MAP (<75 mmHg), SF ratio (<200), ALC (<1500/μL), and RR (≥ 24 times/min), as shown in Table 2. With the new scoring system, we scored each patient and compared the results with the eventual outcomes. For every patient, the total score was derived from the sum of the scores attributed to the variables mentioned above. The minimum and maximum possible scores are 0 and 3 for MPL and 0 and 6 for MSLR. Based on the AUC analysis of the MPL and MSLR scores, both scoring systems have the same sensitivity and specificity, which are 79.1% and 75.5%, respectively (see Figure 2A,B). The AUC of the MPL was 0.802, while the MSLR was 0.807. The cut-off point of the MPL score was ≥2, while the MSLR was ≥4 (see Figure 2C,D). According to the analysis of survival mortality in 30 days using the Kaplan—Meier chart, MPL score ≥ 2 and MSLR score ≥ 4 showed a very significant difference (see Figure 3A,B).

## 4. Discussion

The COVID-19 pandemic has resulted in extensive disruption to healthcare systems, especially in developing countries. In Indonesia, there are still remote areas with limited healthcare facilities that are challenged to promptly diagnose and treat COVID-19 patients. Delays in identifying severe cases and providing more aggressive therapy could lead to increasing death rates. Thus, an easy, quick, and accurate scoring system to determine the severity of COVID-19 pneumonia is needed. Prior to this study, several experts managed to originate and conclude several predictive scores reflecting the specific character of the COVID-19 clinical pathway. Liang et al. arranged a COVID scoring system referring to the cohort criteria of COVID patients in China. Moreover, Bradley et al. did a comparison among CURB-65, NEWS-2, and qSOFA and concluded that NEWS-2 ≥ 5 had a negative predictive score of 98%. The COVID-GRAM critical illness score, which is an analytical score formulated involving several laboratory and clinical parameters, also has a predictive score, but it is no better than NEWS-2. It can be concluded that NEWS-2 is considered a sufficient scoring system. Unfortunately, this scoring system analyzes more respiratory aspects than blood flow dysfunction [24].

Mean arterial pressure (MAP) is the average blood pressure in one cardiac cycle. The minimum MAP required for maintaining organ perfusion needs to be around 60 mmHg. MAP is also an independent predictor of the metabolic syndrome and cardiovascular events, better than systolic blood pressure alone [20,25].

Research by Bansal et al. did a follow-up on critical patients admitted to the ICU and revealed that the total mortality was 45.8%. The mortality rate was significantly lower in patients whose MAP was 70 mmHg (*p* = 0.007). Mortality can be roughly predicted from a low MAP [26,27]. Similar results were obtained by Burstein et al., showing that the crude mortality rate was higher in patients with MAP < 65 mmHg compared to MAP 65–75 mmHg or MAP 75 mmHg (57.0% vs. 29.8% vs. 26.9%, *p* < 0.001 for MAP < 65 mmHg vs. other groups, *p* = 0.36 between groups) [28]. Altschul et al. included MAP ≤ 60 mmHg as one of the variables related to the high mortality rate of COVID-19 in their study [29]. An analysis of the effect of low MAP as one of the risk factors that affect the severity of COVID-19 has also been stated in other studies [16,17,30].

Oxygen saturation is associated with an escalating risk of death. Research by Qi et al. showed that SpO_2_ < 90% highly predicted mortality within 24 h. For every 10% decrease in oxygen saturation, the mortality rate increased by about 2.66 times (*p* = 0.0002; 95% CI OR = 1.45–4.85) [31]. To assess the performance of oxygen perfusion without laboratory facilities, the only parameter that can be used clinically is oxygen saturation (SpO_2_). Oxygen saturation in critically ill patients may reflect microcirculatory conditions, which will also be associated with laboratory markers such as lactate and acid-base deficiency [32]. Imanieh et al. are also in line with this study, integrating oxygen saturation as one of the scoring categories [30].

Du et al. examined COVID-19 patients based on clinical and laboratory criteria and revealed some severe complaints and comorbidities. The five most common complaints in COVID-19 patients were fever (98.9%), dry cough (81.6%), dyspnea (49.7%), fatigue (39.1%), and productive cough (30.7%) [32]. On the other hand, headaches, fatigue, and productive coughs were more common in the survivor group. Patients with shortness of breath and a rapid respiratory rate were more common in the non-survival group (*p* = 0.016) [33]. The same data suggested that severe COVID-19 patients with a respiratory rate of more than 30 times per minute were at risk of having an immediate need for mechanical ventilation along with a poorer prognosis [34]. Research by Nlandu et al. disclosed a difference between the non-survivor groups having a higher respiratory rate [aHR 1.42; 95% CI 1.09–1.92] and a low PaO_2_/FiO_2_ ratio (67.6 [57.9–96.5] vs. 145.5f [73.1–251.2]) [35].

Oxygen supplementation for critically ill patients has been a serious dilemma. Although adequate oxygenation is extremely needed in respiratory failure to maintain microcirculation and tissue function, high doses of oxygen may instead induce toxicity. The study by Kaydu et al. noted that the mean PaO_2_ in the first 24 h in the ICU was 16.2 kPa (122.44 + 31.8 mmHg) and the mean FiO_2_ was 60%. The PaO_2_ was 115.92 ± 46 mmHg in the non-survivor group and 122.07 ± 59.37 mmHg in the survivor group. Statistical analysis showed no difference between these two groups. Data analysis based on the PaO_2_/FiO_2_ group showed insignificant results, which were 218.82 ± 97.81 mmHg for survivors and 213.67 ± 98.9 for non-survivors *p* = 0.583 [36].

However, another study conducted by Eastwood et al. showed significant results in PaO_2_ in critically ill patients [37]. De’Jonge’s study showed that mortality rates increased with high FiO_2_ and low PaO_2_ in the first 24 h in the ICU. The similarity of results between De Longe et al. and Kaydu et al. was in FiO_2_ levels (50.4% vs. 60%) and PaO_2_ values (13.1 kPa vs. 16.2 kPa) in the initial 24 h. These studies also revealed differences in outcomes in critical patient mortality (23% vs. 56%). This discrepancy was due to different research methods in determining the low cutoff PaO_2_ in the first 24 h (the highest alveolar arterial gradient vs. the worst PaO_2_/FiO_2_ ratio) and also to differences in the diagnosis and treatment of patients in the ICU [36,38].

Research involving the COVID-19 population in China showed that, of several laboratory and clinical markers, the independent predictor of ICU admission and mortality was the PaO_2_/FiO_2_ ratio (OR = 0.96, 95% CI: 0.928–0.994, *p* = 0.021). Statistical analysis showed that the AUC was 0.895 (95% CI: 0.826–0.943, *p* < 0.0001), with sensitivity and specificity of 81.2% and 83.1%, respectively, when the cut-off value was 152.86 mmHg [39].

Acute respiratory distress syndrome (ARDS) in COVID-19 patients is a threat leading to a high mortality rate. Acute respiratory distress syndrome (ARDS) criteria that include blood gas analysis raise the question of whether the clinical criteria for saturation ratio and oxygen fraction are comparable markers for respiratory failure. The use of pulse oximetry can reduce the need for blood gas analyzers. Of 329 patients with ARDS (91%) with mechanical ventilation, evaluation in the initial 4 days showed no difference in the clinical characteristics, PF ratio, or SF ratio (92% vs. 88%, *p* = 0.179). There was also no difference in length of stay in the ICU, duration of mechanical ventilation, ventilator-free days, length of hospital stay, or hospital mortality between the two groups [40].

Further studies from Chen et al. stated that the mortality rates of mild, moderate, and severe ARDS according to the Berlin criteria were 37.8%, 36.7%, and 33.7%, respectively (*p* = 0.86). According to the SF ratio criteria, the mortality rates of patients with mild, moderate, and severe ARDS were 30.6%, 23.1%, and 61.1%, respectively (*p* = 0.001). Therefore, these two criteria did not show any differences in clinical characteristics or outcome. The PF ratio was considered insufficient to determine the outcome of ARDS due to its high dependency on the type and strategy of mechanical ventilation. SF ratio criteria in one study was used to determine the severity of ARDS. The results came out promising; therefore, the conclusion stated that the SF ratio prognosticate patients with mild and moderate ARDS better than the PF ratio [40].

Knowledge about the influencing factors of COVID-19 is a key point during treatment decisions. A study that predicted factors behind COVID-19-related mortality showed that neutrophil-to-lymphocyte ratio, absolute lymphocyte count, and high oxygen consumption influence the risk of mortality. The viral pathogenesis of sepsis in COVID-19 is induced initially by lymphocytes. Systemic inflammation by the virus reduces levels of CD4+ T lymphocytes and suppresses levels of CD8+ T lymphocytes, thereby decreasing cellular immunity [41].

The function of lymphocytes as cytotoxic T cells is to maintain immune homeostasis and inflammatory responses to control viral infections. Previous studies reported that functional apoptosis of cytotoxic lymphocytes was associated with the development of viral infections. Although the mechanism of lymphopenia in the course of COVID-19 is still unclear, it is hypothesized that overproduction of pro-inflammatory cytokines due to COVID-19 infection induces strong lymphocyte apoptosis [42].

Basheer et al. showed that the non-survival group had lymphopenia with levels of 1.4 ± 4 vs. 2.06 ± 11 (*p* = 0.001) [39]. In addition, a study comparing the CURB-65 score and pneumonia severity index involving COVID-19 patients for 30 days also showed lymphopenia in the non-survival group. There was a significant difference in lymphopenia in the non-survival and survivor groups: 925 (660–1335) vs. 1325 (970–1752) (*p* = 0.001) [43].

Of the 96 patients we thoroughly followed, the MPL score consisted of 3 points: MAP < 75 (1 point), PF Ratio < 200 (1 point), and lymphocyte absolute < 1500 (1 point), while the MSLR consisted of 6 points: MAP < 75 (1 point), SF Ratio < 200 (1 point), lymphocyte absolute < 1500 (2 points), and respiration rate 24/min (2 points). MPL cutoff is at point 2, while MSLR is at point 4. Both MPL and MSLR have the same sensitivity and specificity (Sensitivity = 79.1%; Specificity = 75.5%). AUC value of MPL vs. MSLR (0.802 vs. 0.807). Thirty-day mortality survival scores for MPL 2 and MSLR 4 were the same (*p* < 0.05).

We obtained these variables after analyzing some of the data from the preliminary research by Asmarawati et al. [17,18]. Based on these factors, we were able to explore that MAP, PF ratio, lymphocyte absolute, SF ratio, and respiratory rate had an independent effect on death. These variables form a scoring system that is able to predict with fairly good sensitivity and specificity. Fortunately, in health facilities where resources are limited, this prompt and accurate scoring system will be accessible.

Various physicians with different fields took part in this study and indirectly underwent randomization. The study population was taken consecutively from the first and second waves of COVID-19, so the characteristics of the population are heterogeneous. The majority of the patients that we followed up on needed blood gas analysis data, making it difficult to perform outside the ICU. Pulse oximetry measurements are affected not only by PaO_2_, but also by pH and venous and arterial oxygen saturation. Low SaO_2_ also indicates arterial hypoperfusion and hypoxemia. In general, this data is a predictive score that can be obtained with lower medical expenses and used to determine the disease’s severity in its early stages. It can be serially compared to reflect the prognosis and enhance patient care.

This study has some limitations. First, age and comorbidities such as diabetes, hypertension, and cardiovascular disease, which are no less important in affecting the severity of COVID-19, were not adjusted accordingly in the scoring system. Second, the sample size was small and only obtained in a single-center study. Thus, further multi-center studies with a larger sample size and better-adjusted analyses are highly anticipated.

## 5. Conclusions

MPL and MSLR scores convey a promising value in predicting the mortality of COVID-19 patients within 30 days. Moreover, these two scoring systems can replace each other because they show the same results. In conclusion, they can help clinicians at hospitals with deficient resources foretell the deterioration and mortality of COVID-19 patients.

## Figures and Tables

**Table 1:** Baseline demographics and clinical characteristics of the subjects.

Demographic Characteristics	n (%) or Mean ± SD
Male	49 (51)
Female	47 (49)
Age (years, Mean ± SD)	52.77 ± 12.28
COVID-19 Severity, n (%)	
Non-critical	23 (24)
Critical	73 (76)
Duration of hospitalization (days, Mean ± SD)	13.72 ± 7.73
Comorbidities, n (%)	
DM	39 (40.6)
HT	32 (33.3)
Heart disease	6 (6.3)
Stroke	2 (2.1)
Outcomes, n (%)	
Death	43 (44.8)
Survive	53 (55.2)
Vital signs, mean ± SD	
Systolic blood pressure (mmHg)	128.2 ± 27.38
Diastolic blood pressure (mmHg)	77.52 ± 15.41
MAP (mmHg)	94.42 ± 18.27
Heart rate (times per minute)	105.5 ± 18.15
Respiration rate (times per minute)	25.59 ± 6.2
Temperature (°C)	36.6 ± 0.6
Oxygen saturation (%)	94.98 ± 4.94
Laboratory parameter, mean ± SD	
Hb (g/dL)	13.1 ± 2.3
Hct (%)	38.5 ± 5.35
Leukocyte (10^3^/μL)	8.88 ± 5.79
Nuetrophill (%)	78.7 ± 15.3
Lymphocyte (%)	12.45 ±11.08
Absolute lymphocyte count (/μL)	1163.8 ± 1498.97
Thrombocyte (10^3^/μL)	263.5 ± 138.86
NLR	6.55 ± 10.99
CRP (mg/L)	20.33 ± 86.11
Procalcitonin (ng/mL)	2.499 ± 10.83
BUN (mg/dL)	14.15 ± 26.37
Creatinine serum (mg./dL)	0.97 ± 2.23
pCO_2_ level (mmHg)	32.07 ± 13.39
HCO_3_ level (mEq/L)	20.4 ± 5.06
PF ratio	147.41 ± 92.41
SF ratio	158.83 ± 84.93

DM—diabetes mellitus; HT—hypertension; ALT—alanine transaminase; AST—aspartate aminotransferase; BUN—blood urea nitrogen; CRP—C-reactive protein; Hb—hemoglobin; HCO_3_-—bicarbonate ion; HCT—hematocrit; MAP—mean arterial pressure; NLR—neutrophil-lymphocyte ratio; pCO_2_—partial pressure of carbon dioxide; PF—PaO_2_/FiO_2_ ratio; SCr—serum creatinine; SF ratio—SpO_2_/FiO_2_ ratio.

**Table 2:** Bivariate and multivariate analysis of MPL and MSLR.

Scoring Category	Death	*p*-Value ^†^	*p*-Value ^‡^	B	SE	B/SE	(B/SE/Cof)	Score
MPL								
MAP < 75 mmHg	15	0.012	0.039	1.19	0.58	2.06 *	1.00	1
PF Ratio < 200	37	0.000	0.003	1.61	0.55	2.92	1.42	1
Lymphocyte absolute < 1500	34	0.001	0.011	1.30	0.51	2.56	1.24	1
MSLR								
MAP < 75 mmHg	15	0.012	0.151	0.84	0.58	1.43 *	1.00	1
SF Ratio < 200	36	0.000	0.129	0.88	0.58	1.52	1.06	1
Lymphocyte absolute < 1500	34	0.001	0.009	1.34	0.52	2.60	1.82	2
RR ≥ 24/min	35	0.000	0.03	1.20	0.55	2.17	1.52	2

MPL—MAP, PF ratio, lymphocyte absolute; MSLR—MAP, SF ratio, lymphocyte absolute, RR; MAP—mean arterial pressure; PF ratio—PaO_2_/FiO_2_; SF ratio—SaO_2_/FiO_2_; RR—respiration rate; B—coefficient beta; SE—standard error; Cof—Coefficient from the lowest value of B/SE; *—coefficient value; **^†^**—*p*-value bivariate; **^‡^**—*p*-value multivariate.

**Table 3:** New score category for predicting mortality in COVID-19 patients.

Scoring Category	Point
MPL	
MAP < 75 mmHg	1
MAP ≥ 75 mmHg	0
PF ratio < 200	1
PF ratio ≥ 200	0
ALC < 1500/μL	1
ALC ≥ 1500/μL	0
MSLR	
MAP < 75 mmHg	1
MAP ≥ 75 mmHg	0
SF ratio < 200	1
SF ratio ≥ 200	0
ALC < 1500/μL	2
ALC ≥ 1500/μL	0
RR ≥ 24/min	2
RR < 24/min	0

MAP—mean arterial pressure; PF ratio—PaO_2_/FiO_2_; SF ratio—SaO_2_/FiO_2_; RR—respiration rate; ALC—absolute lymphocyte count.

## Data Availability

The raw data are available upon request from the corresponding authors.
